# Modeling Research Topics for Artificial Intelligence Applications in Medicine: Latent Dirichlet Allocation Application Study

**DOI:** 10.2196/15511

**Published:** 2019-11-01

**Authors:** Bach Xuan Tran, Son Nghiem, Oz Sahin, Tuan Manh Vu, Giang Hai Ha, Giang Thu Vu, Hai Quang Pham, Hoa Thi Do, Carl A Latkin, Wilson Tam, Cyrus S H Ho, Roger C M Ho

**Affiliations:** 1 Institute for Preventive Medicine and Public Health Hanoi Medical University Hanoi Vietnam; 2 Bloomberg School of Public Health Johns Hopkins University Baltimore, MD United States; 3 Centre for Applied Health Economics Griffith University Brisbane Australia; 4 Griffith Climate Change Response Program Griffith University Brisbane Australia; 5 Odonto Stomatology Research Center for Applied Science and Technology Hanoi Medical University Hanoi Vietnam; 6 Institute for Global Health Innovations Duy Tan University Da Nang Vietnam; 7 Center of Excellence in Evidence-based Medicine Nguyen Tat Thanh University Ho Chi Minh Vietnam; 8 Centre of Excellence in Artificial Intelligence in Medicine Nguyen Tat Thanh University Ho Chi Minh Vietnam; 9 Alice Lee Centre for Nursing Studies Yong Loo Lin School of Medicine National University of Singapore Singapore Singapore; 10 Department of Psychological Medicine National University Hospital Singapore Singapore; 11 Center of Excellence in Behavioral Medicine Nguyen Tat Thanh University Ho Chi Minh Vietnam; 12 Department of Psychological Medicine Yong Loo Lin School of Medicine National University of Singapore Singapore Singapore; 13 Institute for Health Innovation and Technology National University of Singapore Singapore Singapore

**Keywords:** artificial intelligence, applications, medicine, scientometric, bibliometric, latent Dirichlet allocation

## Abstract

**Background:**

Artificial intelligence (AI)–based technologies develop rapidly and have myriad applications in medicine and health care. However, there is a lack of comprehensive reporting on the productivity, workflow, topics, and research landscape of AI in this field.

**Objective:**

This study aimed to evaluate the global development of scientific publications and constructed interdisciplinary research topics on the theory and practice of AI in medicine from 1977 to 2018.

**Methods:**

We obtained bibliographic data and abstract contents of publications published between 1977 and 2018 from the Web of Science database. A total of 27,451 eligible articles were analyzed. Research topics were classified by latent Dirichlet allocation, and principal component analysis was used to identify the construct of the research landscape.

**Results:**

The applications of AI have mainly impacted clinical settings (enhanced prognosis and diagnosis, robot-assisted surgery, and rehabilitation), data science and precision medicine (collecting individual data for precision medicine), and policy making (raising ethical and legal issues, especially regarding privacy and confidentiality of data). However, AI applications have not been commonly used in resource-poor settings due to the limit in infrastructure and human resources.

**Conclusions:**

The application of AI in medicine has grown rapidly and focuses on three leading platforms: clinical practices, clinical material, and policies. AI might be one of the methods to narrow down the inequality in health care and medicine between developing and developed countries. Technology transfer and support from developed countries are essential measures for the advancement of AI application in health care in developing countries.

## Introduction

The first idea of a thinking machine was developed in 1945 when a system that could amplify human knowledge was described in Vannevar Bush’s seminal work [1]. Five years later, Alan Turing mentioned a machine being able to imitate human action and gave chess playing as an example of actions that a computer could do [2]. In 1956, artificial intelligence (AI) was first coined by John McCarthy in a Dartmouth conference [3]. Since then, there have been a few definitions of AI [4-6]. Although there is no consistency in these definitions, one common idea is that AI is an intelligent machine or a system, displaying intelligent behavior.

There are two schools of thought among the AI community: conventional artificial intelligence and computational intelligence [7]. Conventional AI includes machine learning and statistical analysis, while the neural network and fuzzy system belong to computational intelligence [7,8]. Other applications of AI include expert system, automation, and artificial creativity [9]. Expert system and machine learning are two of the most popular applications of AI. The expert system emulates the decision-making ability of humans in a field, while machine learning is a computer program that has the ability to learn from experience. In addition, robotics, a science of dealing with designing and operating robots, with the application of AI, has created robots with improved quality in sensing, vision, and self-awareness [10].

With continuous development and challenges to overcome, AI has been applied in various fields of society such as game playing [11], computer vision [12], speech recognition [13], and expert system in health care [14] and economics [15]. In particular, the contribution of AI in medicine and health care has brought about changes in not only the health system but also patients. The earliest application of AI in medicine dates to 1964, with the corporation of scientists from multidisciplinary research fields for the DENDRAL project [16]. The success of this scientific reasoning is one reason for the explosive spread of AI in biomedicine in the 1970s [17]. Another early application of AI to health care was medical diagnostic decision support systems, which appeared in 1954 [18]. Over the last 60 years, there has been a huge wave of AI technologies in health care. This change is reflected by not only the rapid increase in the number of papers in AI in medicine and health care, but also the appearance of AI in various medical fields [19]. Several AI techniques such as robotics, deep learning, support vector machines, or machine learning have been widely applied in the medical diagnostic system, treatment, and rehabilitation [20-22]. Some scientific publications have shown the effectiveness of AI in medicine and health care. In medical diagnosis, AI has been proved to be effective in improving the diagnostic accuracy for physical diseases [23-25]. The expert system has been used for diagnosis of diseases such as heart disease [26] and diabetes [27] and has proven to be useful for diagnosis and basic treatment advice [27]. For mental illness, AI may be useful for psychiatric consultations. Machine learning has been applied in a predictive model, which could identify patients with symptoms of schizophrenia and attempting to commit suicide with 74% and 80%-90% accuracy, respectively [28,29]. In terms of treatment, most robots assist clinicians in surgery but do not independently perform operations [30].

Due to the variety of AI applications in medicine and health care, there is a need to understand the current states of AI applications, major topics, and the research area of AI in medicine and health care and to identify research gaps. This study attempted to contribute to this understanding by analyzing the context and landscape of research topics [19]. Compared with previous scientometrics research, this study is global and assessed a wide range of AI utilities in medicine and health care [31,32]. Our study used scientific publications downloaded from the Web of Science to model the change and achievement of research topics and landscape in AI applications in health and medicine documents.

Thus, this study evaluated the global development of scientific publications from 1977 to 2018 and characterized research landscapes and constructs of disciplines applied to AI in medicine and health care. By decoding these patterns, we can effectively explore the changes in the growth of publications and may therefore provide better information for other researchers and policymakers in priority settings and evaluation.

## Methods

### Search Strategies and Data Source

The full strategy of our study has been presented elsewhere [33] (Multimedia Appendix 1). Data were retrieved from the Web of Sciences database provided by Thomson Reuters Institute for Scientific Information. We chose this database because of its outstanding advantages over other databases such as Scopus or PubMed: It contains bibliographic data since 1900, has a higher scientific journal impact, has more indexes, and is better in representing metadata [34].

### Data Download

The data under .txt format, including the paper information (publication name, authors, journals’ name, year of publication, keywords, author affiliations, total citation, subject research, and abstracts), were downloaded from Web of Science. Two researchers worked independently to simultaneously download the data. Subsequently, we filtered all downloaded data by excluding papers that were published in 2019, not original articles and reviews, written by an anonymous author, and not in English (Multimedia Appendix 2). Any conflict was resolved by discussion. All the data were merged and analyzed by STATA software (STATACorp LLC, College Station, TX).

### Data Analysis

We analyzed data based on the characteristic of publication (total papers, publication years, and number of papers by countries), research areas, abstracts (terms and contents of the abstract), citations, and usages (number of downloads). Subsequently, we used STATA software to perform a content analysis of the abstracts. We applied principal component analysis to identify the landscape of AI in medicine and health care. The Jaccard similarity index was utilized to identify research topics or terms most frequently co-occurring with each other. We applied a topic modeling technique for data mining and determining relationships among text documents. Specifically, we chose latent Dirichlet allocation (LDA), which is one of the most popular methods in this field for further analysis. LDA was a helpful technique to classify papers into similar topics [35-39]. It helps recognize the structure of research development, current trends, and interdisciplinary landscapes of research in AI applied to medicine. Using LDA, we classified text in each abstract to a topic where Dirichlet is used as a distribution over discrete distribution; each component in a random vector is the probability of drawing the words/texts associated with that component. Principle component analysis (PCA) was used to classify the research disciplines into corresponding groups.

Thus, by applying LDA, we could obtain an in-depth view of the trends of AI in health care and annotate the documents’ topic to discover hidden themes [40]. Additionally, the landscape analysis addressed the relationship between research disciplinaries and showed how research areas in medicine and health care changed due to AI. The summary of analytical techniques for each data types is presented in Table 1.

**Table 1:** Summary of analytical techniques for each data types.

Type of data and unit of analysis		Analytical methods		Presentations of results
**Abstracts**				
	Words	Frequency of co-occurrence		Number of papers by countries in abstracts
	Papers	Latent Dirichlet allocation		Ten classifications of research topics
**Web of Science classification of research areas**				
	Web of Science research areas	Coincidence analysis		Dendrogram of research disciplines (Web of Science classification) The Web of Science research areas constructing Latent Dirichlet allocation research topics

## Results

### Number of Published Items and Publication Trend

As seen in Table 1, the number of AI publications increased rapidly during the past 40 years. Notably, most of the publications (23,216 papers, 84.6%) were published during the last 10 years, and 60.6% of the total citation belonged to this period. The usage of papers was counted by the number of downloads. The mean use rate (download rate) within the last 6 months, of papers published in the year 2018 was three times higher than that of papers published in the previous years. The mean use rate within the last 5 years reached its peak in 2013 and decreased from 2012.

We analyzed the frequency of a country where the study was conducted, which was mentioned in the abstract (Table 2). Among 50 countries, the United States appeared the most (1867 times, 40.4%). Notably, only four African countries (Egypt, Niger, Kenya, and Nigeria) were mentioned in the abstracts. In addition, 13 Asian countries contributed to this list, and two Asian leaders of AI technologies—China (including Taiwan and Hong Kong) and India—accounted for 9.8% and 4.32% of the total papers, respectively.

### Research Landscapes

**Figure 1:**
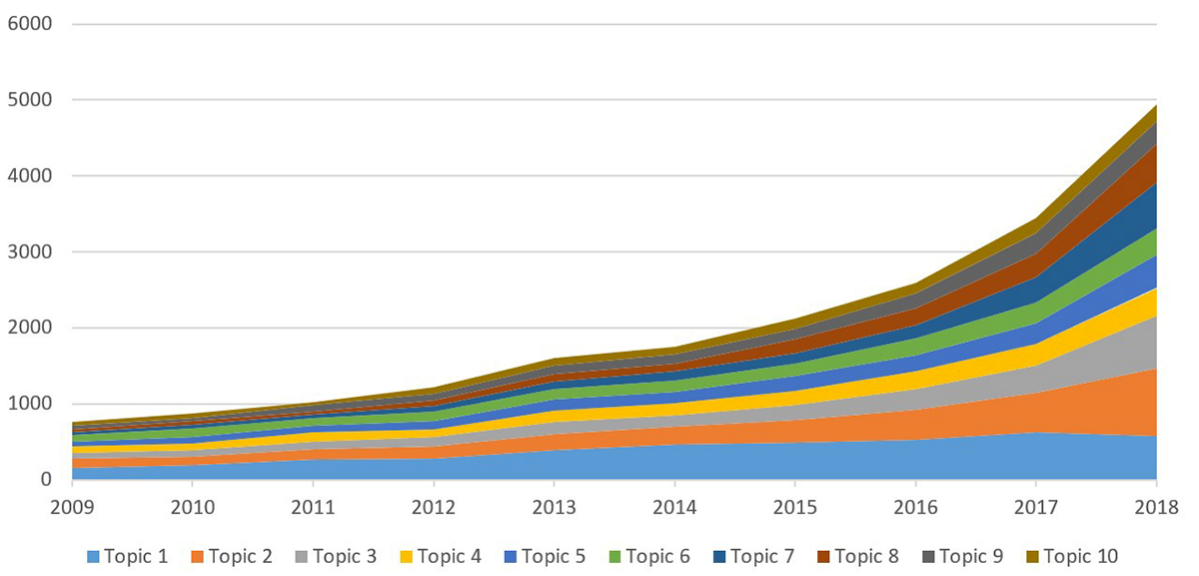
Changes in the applications of artificial intelligence to health and medicine in the past 10 years.

Table 3 presents the scientific research topics constructed by LDA. By analyzing the most frequent words and titles, we could manually annotate the label of each topic. Robotics, which most mentioned the 10 topics and branches of AI (topic 1, topic 6, and topic 9), has supported surgery and treatment. AI types were applied the most in the diagnosis and prediction (topic 2, topic 5, and topic 7). Based on development visualization, there was a growing trend in some of the 10 topics, with different rates. The number of papers related to topic 1 was highest and increased gradually but with a slower rate in recent years. Moreover, the number of papers in topic 2 and topic 3 increased at a higher rate than that of papers in other topics (Figure 1).

**Table 2:** General characteristics of publications.

Year published	Total number of papers	Total number of citations	Mean citation rate per year	Total usage in last 6 months	Total usage in last 5 years	Mean use rate in last 6 months	Mean use rate in last 5 years
2018	5619	7084	1.26	35,677	57,370	6.35	2.04
2017	3919	19,639	2.51	11,320	56,890	2.89	2.90
2016	2969	27,636	3.10	6684	55,729	2.25	3.75
2015	2416	31,168	3.23	4,193	46,820	1.74	3.88
2014	1990	30,523	3.07	2473	35,563	1.24	3.57
2013	1839	35,259	3.20	2010	37,934	1.09	4.13
2012	1385	30,130	3.11	1114	19,950	0.80	2.88
2011	1189	37,313	3.92	1379	18,603	1.16	3.13
2010	1010	28,270	3.11	661	10,185	0.65	2.02
2009	880	27,847	3.16	678	9607	0.77	2.18
2008	718	26,865	3.40	530	6944	0.74	1.93
2007	557	19,402	2.90	343	4575	0.62	1.64
2006	479	24,213	3.89	375	4923	0.78	2.06
2005	367	13,460	2.62	178	2473	0.49	1.35
2004	350	16,294	3.10	216	3240	0.62	1.85
2003	262	14,671	3.50	188	2465	0.72	1.88
2002	195	14,143	4.27	157	2109	0.81	2.16
2001	191	8852	2.57	117	1766	0.61	1.85
2000	170	8056	2.49	87	1171	0.51	1.38
1999	150	5517	1.84	61	678	0.41	0.90
1998	163	4396	1.28	44	606	0.27	0.74
1997	124	7179	2.63	89	877	0.72	1.41
1996	114	3310	1.26	29	373	0.25	0.65
1995	98	3182	1.35	38	334	0.39	0.68
1994	100	3570	1.43	37	328	0.37	0.66
1993	61	2238	1.41	29	222	0.48	0.73
1992	62	1395	0.83	25	225	0.40	0.73
1991	41	683	0.59	31	101	0.76	0.49
1990	8	179	0.77	5	16	0.63	0.40
1989	2	438	7.30	2	9	1.00	0.90
1988	7	117	0.54	3	21	0.43	0.60
1987	6	18	0.09	2	8	0.33	0.27
1986	5	59	0.36	4	14	0.80	0.56
1985	2	4	0.06	0	1	0.00	0.10
1984	1	7	0.20	0	3	0.00	0.60
1980	1	51	1.31	0	7	0.00	1.40
1977	1	3	0.07	1	4	1.00	0.80

**Table 3:** Number of papers by countries as study settings.

Rank	Country settings	Frequency, n (%)
1	United States	1867 (40.4)
2	Ireland	332 (7.2)
3	Taiwan	215 (4.7)
4	China	208 (4.5)
5	United Kingdom	194 (4.2)
6	India	181 (3.9)
7	Japan	164 (3.6)
8	Australia	141 (3.1)
9	Canada	86 (1.9)
10	Iran	83 (1.8)
11	Germany	81 (1.8)
12	Italy	74 (1.6)
13	Brazil	58 (1.3)
14	Spain	56 (1.2)
15	France	55 (1.2)
16	Sweden	43 (0.9)
17	Turkey	43 (0.9)
18	Israel	37 (0.8)
19	New Zealand	33 (0.7)
20	Wallis and Futuna	31 (0.7)
21	Hong Kong	27 (0.6)
22	Mali	27 (0.6)
23	Netherlands	25 (0.5)
24	Poland	25 (0.5)
25	Singapore	23 (0.5)
26	Switzerland	22 (0.5)
27	Greece	21 (0.5)
28	South Africa	20 (0.4)
29	Saudi Arabia	19 (0.4)
30	Malaysia	18 (0.4)
31	Egypt	17 (0.40
32	Pakistan	17 (0.4)
33	Denmark	13 (0.3)
34	Belgium	12 (0.3)
35	Georgia	12 (0.3)
36	Niger	12 (0.3)
37	Kenya	11 (0.2)
38	Mexico	11 (0.2)
39	Nigeria	11 (0.2)
40	Austria	10 (0.2)
41	Finland	10 (0.2)
42	Chile	9 (0.2)
43	Norway	9 (0.2)
44	Portugal	9 (0.2)
45	Thailand	9 (0.2)
46	United Arab Emirates	9 (0.2)
47	Colombia	8 (0.2)
48	Jordan	8 (0.2)
49	Serbia	8 (0.2)
50	Czech	7 (0.2)

**Figure 2:**
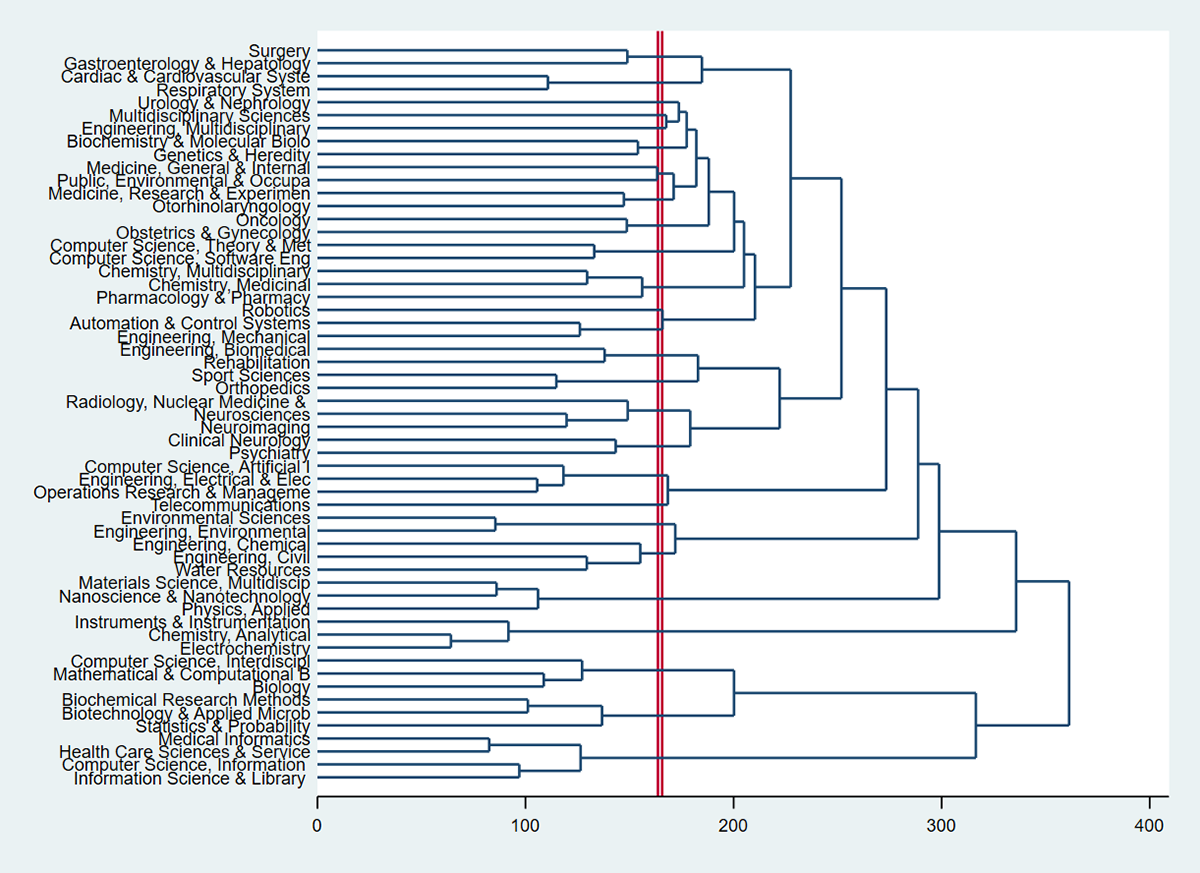
Dendrogram of coincidence of research areas using the Web of Science classifications.

Based on the classification of research areas in the Web of Science, we identified the dendrograms for the areas (Figure 2). The dendrogram includes the clades and leaves. The clade is the branch, and each clade includes one or more research areas. The horizontal axis shows the distance or dissimilarity between research areas. Each joining (fusion) of two clusters is represented on the diagram by the splitting of a vertical line into two vertical lines. The vertical position of the split, shown by a short bar, gives the distance (dissimilarity) between the two research areas. It shows that the AI applications focused on seven following research areas: surgery, robotics, and noncommunicable diseases (hepatocardiac disorders or cancer); neurosciences and psychiatry; the application of electronic health (telecommunication); chemical sciences; nanoscience; electrochemistry; and medical informatics and biotechnology. It seems that AI in medicine was assigned mainly to the disciplines diseases and treatment (surgery or robotics application).

**Figure 3:**
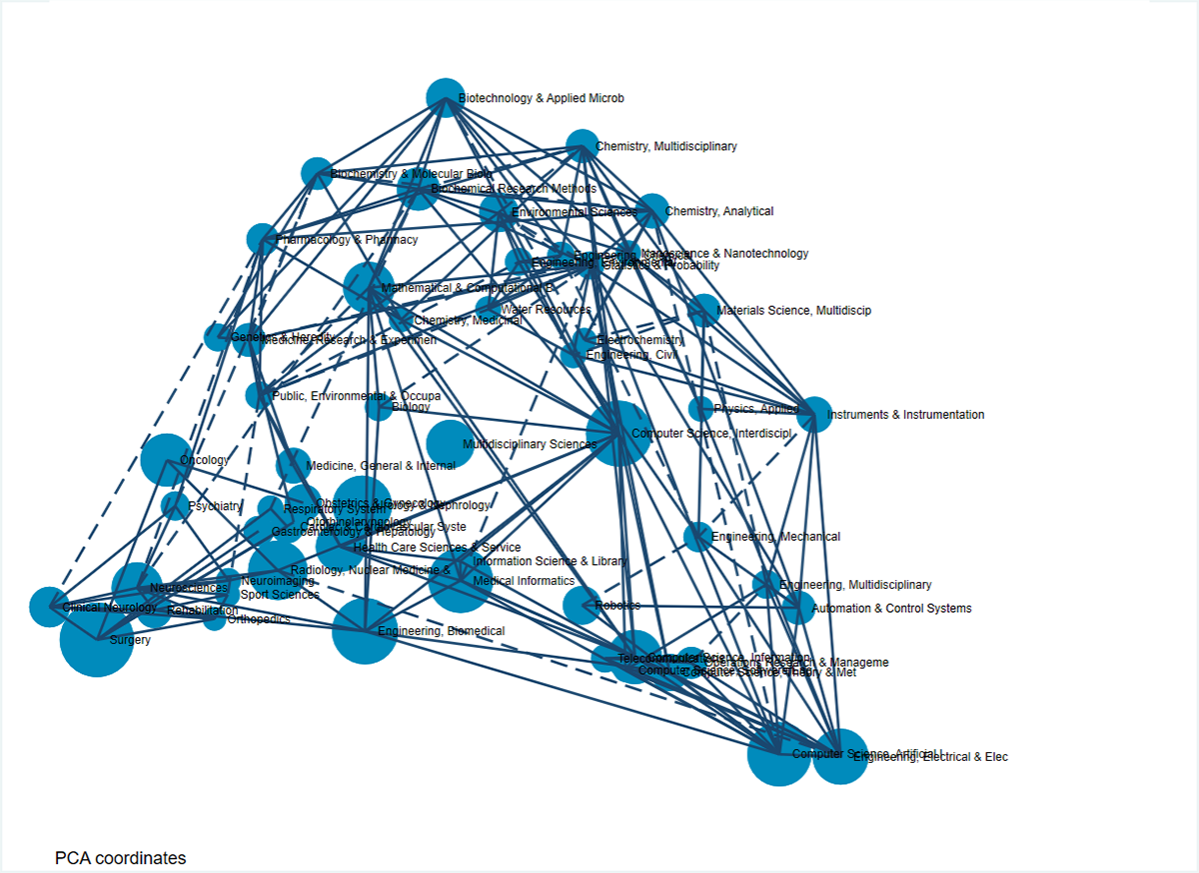
Landscapes of artificial intelligence in medicine by Web of Science categories. PCA: principal component analysis.

We applied PCA to identify the landscape of AI in medicine and health care (Figure 3). Based on the size of the node, most papers belonged to the following research categories: clinical: surgery, radiology, and nuclear medicine; technology: biomedical, robotics, computer science, medical informatics; and diseases: oncology, general and internal medicine and noncommunicable diseases. As shown in Figure 2, a strong relationship among the applications of AI in treatment, diseases, and medical informatics shows that AI assisted surgeons, especially in some diseases for which surgery is key in treatment or diagnosis, such as cancer or cardiovascular diseases. The combination of information science, computer science, and health care, called health informatics, has created a wide range of applications, from cell level to population level [41]. Collision of several computer science–related fields and medical fields created a multidisciplinary science, which has led to better chances of providing the best treatment to patients. Additionally, the development of computer sciences has contributed to the advancement of AI in pharmacy, biotechnology, and chemistry in areas such as drug discovery, drug identification and validation, and drug trials.

We compared ten research topics by LDA (Table 4) with Web of Science research areas (Multimedia Appendices 3-5) to identify the consistency of research disciplinaries of AI in medicine and health care. Computer science and its related fields appeared the most (eight topics). The major application of computer science has been in medical fields: from cells (gene microbiology information, topic 5), disease (oncology, cardiovascular, topic 7), and diagnosis and treatment (topic 1, topic 6, and topic 9) to health policy (topic 3). Additionally, AI types were used the most in medicine and health care, including expert systems, artificial neural networks, machine learning, and natural language processing. Robot and surgery were two applications mentioned the most in topic 1, topic 6, and topic 9. Robotic-assisted procedures were used for cancer surgery and cardiovascular diseases. Robot-assisted therapy was used in treating sports concussion or neurorehabilitation (topic 9).

**Table 4:** Ten research topics classified by latent Dirichlet allocation.

Latent Dirichlet allocation topics	Frequency, n (%)	Topic name
Topic 1	4,3524, 4352 (18.1)	Comparative evaluation of robot-assisted surgery
Topic 2	3662 (15.2)	Expert system for diseases diagnosis and prediction
Topic 3	2839 (11.8)	Health system and policy on AIs^a^ in medicine
Topic 4	2182 (9.1)	Artificial neural networks in treatment selection
Topic 5	2089 (8.7)	AI-based gene and protein analysis and prediction
Topic 6	2065 (8.6)	Precision robotics and personalized medicine
Topic 7	2008 (8.4)	Enhanced diagnosis and classification by AI-based images analysis
Topic 8	1922 (8.0)	Using machine learning to predict risk, disease progression, and treatment outcomes
Topic 9	1564 (6.5)	Robot-assisted rehabilitation treatment
Topic 10	1350 (5.6)	Natural language processing tools for biomedical texts and clinical notes

^a^AI: artificial intelligence.

## Discussion

### Principal Results

By analyzing scientific research publications and modeling their research topics, we generally described the 42-year development and identified the trend of AI application in medicine and health care. The mean use rate related to the application of AI in medicine was the highest in the last 5 years and tended to reduce since 2012. This can be explained by the rapid development of technology and research [42]: Scientific papers published more than 5 years ago would not attract the attention of scientists. Therefore, dissemination efforts need to be taken into consideration by not only policy makers but also authors, to increase the influence and implement changes in practice settings [43]. In addition, the results show a rapid increase in research productivity and downloaded papers in the last 5 years. Its growth was contributed mostly by western countries, driven by the United States. Among 11 Asian countries in that list, China and India were two leaders in research on AI in medicine. The application of AI has benefitted the health care system in high-income countries. One study showed that the United States could save US $5-$8 billion per year with the application of information technology in health care [44]. Another recent analysis found that with the application of AI, we can save up to US $150 billion in yearly health costs [45]. AI, however, has not been widely used in low-income countries. This could be due to the undeveloped infrastructure in the internet, technology, and health systems and a lack of highly qualified human resource. Regardless of the disadvantages, AI holds promise for changing health care services in low-income countries [46].

Based on the topics and research areas, we found that the application of AI in medicine and health care has been focused on robot support in surgery (topic 1) and rehabilitation (topic 9), AI in diagnosis and clinical decisions support (topic 2, topic 4, topic 5, topic 7, topic 8, and topic 10), and AI in health care system management (topic 3). First, for clinical treatment, our results confirm that medical robots and robot-assisted surgeries have been widely used [47,48]. AI has been widely applied in surgery due to its benefits for patients and medical professionals, such as increased accuracy, reduced operation time, minimized surgical trauma, and reduced length of recovery time for patients [49]. Second, AI methods such as machine learning and natural language process analyze complex medical data [20], decrease time spent finding relevant evidence, and reduce medical errors that improve the quality of diagnosis in medical health care [50]. Finally, AI will certainly be applied more in the health care system in the future owing to its advantages over the traditional decision-making process. On the other hand, the fact that users do not know how the results are analyzed by the “black box” algorithms, ethnic differences in validity of facial recognition technology for genetic diagnosis, medical and behavioral conditions [51,52], and ethnic bias in training data set [53] raise questions about product liability, privacy and data protection, and ethical and legal issues [51]. Thus, researchers have voiced their concern about legacy and ethical guidelines that are lagging behind the development of AI in health care and medicine [51].

### Future Implications

Our findings have some implications for health research and policy. The quick development of AI applications in health and medicine requires some preparations. AI may change the relationship between caregivers and patients, as the direct interaction might reduce due to digital tools such as a free app in the patient’s personal device, which could diagnose the disease in some cases or even lead to self-diagnosis via the Web [54]. Thus, it is necessary for all parties involved to ensure that, in the case of mental health diagnosis, for instance, subtle signs of mental illness would not be neglected [55]. In addition, standard guidelines or laws about collecting private information or application of AI in all health care sectors are urgently needed [56], as the application of AI in health care and medicine has potential threats to patients’ privacy and safety. Finally, AI is transforming health care in resource-poor settings and reducing the gap between rural and urban areas [46]. In rural areas of developing countries, the shortage of medical doctors and trained nurses and the limitation of medical techniques and machines have reduced the quality of medical services [57]. In addition, it is difficult to attract skilled medical workers in rural areas due to the poor working environment and living conditions [58]. However, the development of AI applications can be a solution to these problems. For instance, the AI method (machine learning) proposed a model helping forecast dengue outbreaks in China [59]. In addition, AI has proven to be effective, with a high accuracy of breast cancer detection [60,61]. Moreover, AI can reduce medical costs in developing countries. For example, a highly effective AI method could provide an alternative to expensive diagnostic methods to classify acute leukemia [62]. However, absorptive capacity, local culture, legacy [63], and infrastructure (eg, electricity, internet, or financial source) should be carefully taken into consideration [64]. Notably, policy development for AI should be given more attention, since its failure has been recognized in developing countries such as Vietnam [65].

### Limitations

Our study has several limitations worth noting. First, we choose only Web of Science as the database, which may not cover all the publications in the research fields. Second, only English articles and reviews were analyzed in this study. Finally, we applied LDA to model the topic research in title and abstracts, not the full text. However, two other methods (coincidence analysis and PCA) confirmed similar results about the connections of research topics. Thus, LDA could be considered a support method to reduce the workload in the screening step for future systematic reviews [66].

### Conclusions

The application of AI in medicine has grown rapidly and focuses on three leading platforms: clinical practices, clinical material, and policies. AI might be one of the methods to reduce the inequality in health care and medicine between developing and developed countries. Technology transfer and support from developed countries, along with the internal efforts of poor-setting countries, help in the development of AI applications in health care and medicine.

## Appendix

Multimedia Appendix 1 Search results (Web of Science).

Multimedia Appendix 2 Selection of papers in the Web of Science database.

Multimedia Appendix 3 The Web of Science research areas constructing latent Dirichlet allocation research topics (topics 1-3).

Multimedia Appendix 4 The Web of Science research areas constructing latent Dirichlet allocation research topics (topics 4-6).

Multimedia Appendix 5 The Web of Science research areas constructing latent Dirichlet allocation research topics (topics 7-10).

## Abbreviations

AI: artificial intelligence
LDA: latent Dirichlet allocation
PCA: principal component analysis
