# Supplementary material for: Modeling Research Topics for Artificial Intelligence Applications in Medicine: Latent Dirichlet Allocation Application Study
Source: J Med Internet Res. 2019 Nov 1;21(11):e15511. doi: 10.2196/15511 (PMC6858616; doi:10.2196/15511)
Supplement: Multimedia Appendix 1 [file jmir_v21i11e15511_app1.pdf]

Supplementary

Table S1. Search results (WOS)

Number	Search query	Result
#1	TS=("Artificial intelligence" OR "Machine intelligence" OR "artificial neural network*" OR "Machine learning" OR "Deep learn*" OR "Natural language process*"OR "Robotic*" OR "thinking computer system" OR "fuzzy expert system*" OR "evolutionary computation" OR "hybrid intelligent system*")	332,892
#2	TS=(disease* OR illness OR health-related OR medic* OR "medical diagnosis" OR treatment OR health* OR wellness OR well-being)	10,609,911
#3	#2 AND #1	44,519
#4	#2 AND #1<br>Refined by: [excluding] PUBLICATION YEARS: (2019)	43,768
#5	#2 AND #1<br>Refined by: [excluding] PUBLICATION YEARS: (2019) AND [excluding] DOCUMENT TYPES: (PROCEEDINGS PAPER OR BOOK CHAPTER OR MEETING ABSTRACT OR EDITORIAL MATERIAL OR LETTER OR NEWS ITEM OR BOOK OR BOOK REVIEW OR CORRECTION OR DATA PAPER OR EARLY ACCESS OR NOTE OR REPRINT OR BIOGRAPHICAL ITEM OR RETRACTED PUBLICATION OR DATABASE REVIEW)	28,344
#6	#2 AND #1<br>Refined by: [excluding] PUBLICATION YEARS: (2019) AND [excluding] DOCUMENT TYPES: (PROCEEDINGS PAPER OR BOOK CHAPTER OR MEETING ABSTRACT OR EDITORIAL MATERIAL OR LETTER OR NEWS ITEM OR BOOK OR BOOK REVIEW OR CORRECTION OR DATA PAPER OR EARLY ACCESS OR NOTE OR REPRINT OR BIOGRAPHICAL ITEM OR RETRACTED PUBLICATION OR DATABASE REVIEW) AND [excluding] LANGUAGES: (GERMAN OR SPANISH OR FRENCH OR CHINESE OR PORTUGUESE OR TURKISH OR RUSSIAN OR KOREAN OR ITALIAN OR POLISH OR CZECH OR HUNGARIAN OR CROATIAN OR JAPANESE OR SLOVENIAN OR CATALAN OR ROMANIAN OR UKRAINIAN OR MALAY OR SLOVAK)	27,617
#7	AU=Anonymous	1,406,134
#8	#6 NOT #7	27,607
#9	#8 Refined by: [excluding] Undefined data	27,451
