# Supplementary figures and images for: Modeling Research Topics for Artificial Intelligence Applications in Medicine: Latent Dirichlet Allocation Application Study
Source: J Med Internet Res. 2019 Nov 1;21(11):e15511. doi: 10.2196/15511 (PMC6858616; doi:10.2196/15511)

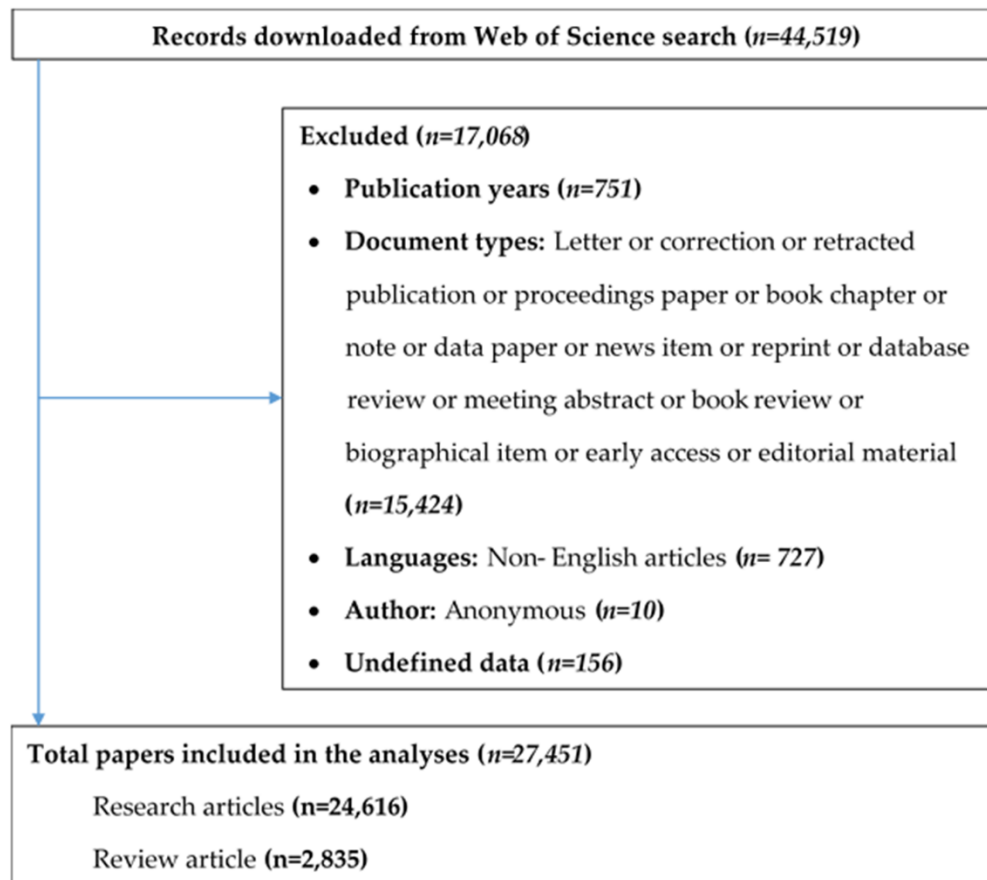

**Figure S1.** Selection of papers in the Web of Science database

Supplement: Multimedia Appendix 2 [file jmir_v21i11e15511_app2.pdf]
