# Supplementary material for: Modeling Research Topics for Artificial Intelligence Applications in Medicine: Latent Dirichlet Allocation Application Study
Source: J Med Internet Res. 2019 Nov 1;21(11):e15511. doi: 10.2196/15511 (PMC6858616; doi:10.2196/15511)
Supplement: Multimedia Appendix 3 [file jmir_v21i11e15511_app3.pdf]

Table S2. The WOS research areas constructing LDA research topics (Topics 1-3)

No	Category	Topic 1	Category	Topic 2	Category	Topic 3
	Total	4,349	Total	3,662	Total	2,838
1	Surgery	37.3%	Computer Science, Artificial	21.0%	Computer Science, Artificial	6.1%
2	Urology & Nephrology	27.7%	Engineering, Electrical	13.8%	Computer Science, Information	5.3%
3	Oncology	14.2%	Computer Science, Interdisciplinary	12.7%	Medical Informatics	4.5%
4	Obstetrics & Gynecology	10.3%	Engineering, Biomedicine	11.6%	Engineering, Electrical	4.0%
5	Otorhinolaryngology	5.7%	Medical Informatics	9.9%	Health Care Sciences	3.8%
6	Gastroenterology & Hepatology	4.8%	Computer Science, Information	7.7%	Computer Science, Interdisciplinary	3.3%
7	Cardiac & Cardiovascular	4.5%	Mathematical & Computational	6.8%	Surgery	3.1%
8	Respiratory System	3.0%	Computer Science, Theory	5.5%	Engineering, Biomedicine	3.0%
9	Medicine, General & Internal	3.0%	Operations Research & Management	4.9%	Robotics	3.0%
10	Radiology, Nuclear Medicine	1.7%	Instruments & Instrumentation	4.3%	Computer Science, Theory	2.7%
