# Supplementary material for: Modeling Research Topics for Artificial Intelligence Applications in Medicine: Latent Dirichlet Allocation Application Study
Source: J Med Internet Res. 2019 Nov 1;21(11):e15511. doi: 10.2196/15511 (PMC6858616; doi:10.2196/15511)
Supplement: Multimedia Appendix 4 [file jmir_v21i11e15511_app4.pdf]

Table S3. The WOS research areas constructing LDA research topics (Topics 4-6)

No	Category	Topic 4	Category	Topic 5	Category	Topic 6
	Total	2,182	Total	2,089	Total	2,062
1	Computer Science, Artificial	11.0%	Biochemical Research	8.5%	Radiology, Nuclear Medicine	8.9%
2	Engineering, Biomedical	5.5%	Mathematical & Computational Biology	6.7%	Engineering, Biomedicine	7.1%
3	Engineering, Chemical	5.1%	Biotechnology & Applied Microbiology	6.5%	Robotics	6.5%
4	Water Resources	4.5%	Biochemistry & Molecular biology	5.8%	Surgery	6.1%
5	Chemistry, Analytical	3.5%	Multidisciplinary Science	5.5%	Engineering, Electrical	4.4%
6	Medical Informatics	2.6%	Genetics & Heredity	4.6%	Materials Science, Multidisciplinary	4.1%
7	Materials Science, Multidisciplinary	2.6%	Computer Science, Interdisciplinary	3.9%	Automation & Control	3.2%
8	Computer Science, Interdisciplinary	2.4%	Chemistry, Multidisciplinary	3.3%	Engineering, Mechanic	3.0%
9	Engineering, Civil	2.1%	Chemistry, Medicinal	3.3%	Oncology	2.9%
10	Chemistry, Multidisciplinary	2.0%	Pharmacology & Pharmacy	3.0%	Instruments & Instrumentation	2.6%
