# Supplementary material for: Modeling Research Topics for Artificial Intelligence Applications in Medicine: Latent Dirichlet Allocation Application Study
Source: J Med Internet Res. 2019 Nov 1;21(11):e15511. doi: 10.2196/15511 (PMC6858616; doi:10.2196/15511)
Supplement: Multimedia Appendix 5 [file jmir_v21i11e15511_app5.pdf]

Table S4. The WOS research areas constructing LDA research topics (Topics 7-10)

	Category	Topic 7	Category	Topic 8	Category	Topic 9	Category	Topic 10
No	Total	2,008	Total	1,922	Total	1,564	Total	1,350
1	Radiology, Nuclear Medicine	13.7%	Medical Informatics	5.7%	Rehabilitation	9.2%	Medical Informatics	14.2%
2	Neurosciences	7.6%	Health Care Sciences	4.0%	Neurosciences	8.6%	Computer Science, Interdisciplinary	9.2%
3	Engineering, Biomedicine	7.1%	Multidisciplinary Science	3.4%	Engineering, Biomedicine	7.0%	Computer Science, Information	8.4%
4	Computer Science, Interdisciplinary	4.6%	Medicine, General & Internal	2.9%	Clinical Neurology	4.4%	Health Care Sciences	8.3%
5	Neuroimaging	3.2%	Computer Science, Interdisciplinary	2.9%	Sport Sciences	3.0%	Information Science & Library	4.7%
6	Clinical Neurology	3.2%	Psychiatry	2.8%	Robotics	2.4%	Mathematical & Computational Biology	3.2%
7	Computer Science, Artificial intelligence	3.2%	Oncology	2.5%	Surgery	1.7%	Computer Science, Artificial	2.3%
8	Engineering, Electric	3.0%	Engineering, Biomedicine	2.4%	Multidisciplinary Science	1.7%	Engineering, Biomedicine	1.6%
9	Medical Informatics	2.8%	Urology & Nephrology	2.2%	Medical Informatics	1.2%	Biochemical Research	1.2%
10	Multidisciplinary Science	2.7%	Surgery	2.2%	Computer Science, Artificial	1.2%	Multidisciplinary Science	1.1%
